# Supplementary material for: Control of cell death/survival balance by the MET dependence receptor
Source: eLife. 2020 Feb 24;9:e50041. doi: 10.7554/eLife.50041 (PMC7039684; doi:10.7554/eLife.50041)
Supplement: Supplementary file 1. [file elife-50041-supp1.docx]

| **Key Resources Table** | | | | |
| --- | --- | --- | --- | --- |
| **Reagent type (species) or resource** | **Designation** | **Source or reference** | **Identifiers** | **Additional information** |
| genetic reagent (Mouse) | Mouse C57/bl6 MET D1374N | Developed by the Mouse Clinical Institute of Strasbourg, France | Homemade |  |
| cell line (Human) | HEK293 | ATCC | #CRL-11268  RRID:CVCL_1926 | Human embryonic kidney |
| cell line  (Human) | MCF10A | ATCC | #CRL-10317 RRID:CVCL_0598 | Human mammary epithelial cells |
| cell line  (Human) | BMEL - Bipotential mouse embryonic liver | Homemade | Homemade | Harvested from E14 mouse embryos of C57/bl6 WT orMET D1374 |
| cell line  (Human) | IHH | Dr F. Kuipers (Groningen, The Netherlands) |  | Immortalized human hepatocytes |
| transfected construct (Human cDNA) | pEGFP-C3 | Clontech |  | Expression vector for p40MET |
| antibody | Anti-FAS antibody;Monoclonal; Mouse | Becton Dickinson | Jo-2  #554254  RRID:AB_395326 | Injection in mice 4 µg/20 g |
| antibody | Anti-MET cytoplasmic domain; Monoclonal; Mouse | Life Technology | 3D4  #37-0100  RRID:AB_2533289 | 1/1000 |
| antibody | Anti-MET C-terminal domain; Monoclonal; Mouse | Cell Signaling Technology | #3148S  RRID:AB_1031042 | 1/1000 |
| antibody | Anti-phospho-MET (Y1234/1235); Polyclonal; Rabbit | Cell Signaling Technology | #3126  RRID:AB_331713 | 1/1000 |
| antibody | Anti- phospho-ERK (Thr202/Tyr204); Monoclonal; Mouse | Cell Signaling Technology | #9106  RRID:AB_331768 | 1/2000 |
| antibody | Anti-phospho-AKT (Ser-473); Monoclonal, Mouse | Cell Signaling Technology | #9271  RRID:AB_329825 | 1/1000 |
| antibody | Anti-MCU; Polyclonal; Rabbit | Cell Signaling Technology | D2Z3B  #14997  RRID:AB_2721812 | 1/1000 |
| antibody | Anti-cleaved Caspase 3(asp175); Polyclonal; Rabbit | Cell Signaling Technology | #9661  RRID:AB_2341188 | 1/1000 IHC and WB  1/250 IF |
| antibody | Anti-cytochrome C; Monoclonal, Mouse | BD Biosciences | 20E8 | 1/250 IF |
| antibody | Anti-calnexin; polyclonal; rabbit | Abcam | #ab75801 RRID:AB_1310022 | 1/2000 |
| antibody | Anti- FACL4/ACSL4; Polyclonal. Rabbit | Novus Biologicals | #NBP2-16401 | 1/1000 WB  1/500 IF |
| antibody | Anti-GFP; Polyclonal; Rabbit | Sigma | #G1544  RRID:AB_439690 | 1/1000 |
| antibody | Anti- PARP-1; Polyclonal; Rabbit | Santa Cruz Biotechnology | H250  #sc-7150  RRID:AB_2160738 | 1/1000 |
| antibody | Anti- GAPDH; Monoclonal; Mouse | Santa Cruz Biotechnology | 6C5  #sc-32233  RRID:AB_627679 | 1/10000 |
| antibody | Anti-ERK2; Polyclonal; Rabbit | Santa Cruz Biotechnology | C14  #sc-154  RRID:AB_2141292) | 1/1000 |
| antibody | Anti-AKT; Polyclonal; Rabbit | Santa Cruz Biotechnology | #sc-8312  RRID:AB_671714 | 1/1000 |
| antibody | Green-fluorescent Alexa fluor 488 conjugated anti-mouse IgG | Invitrogen Molecular Probes | #A11029  RRID:AB_138404 | 1/1000 |
| antibody | Red-fluorescent Alexa fluor 594 conjugated anti-rabbit IgG | Invitrogen Molecular Probes | #A11012  RRID:AB_141359 | 1/1000 |
| antibody | Peroxidase-coupled anti-mouse IgG(H/L) | Jackson Immunoresearch Laboratories | #115-035-146  RRID:AB_2307392 | 1/10000 |
| antibody | Peroxidase-coupled anti-rabbit IgG(H/L) | Jackson Immunoresearch Laboratories | #711-035-152  RRID:AB_10015282 | 1/10000 |
| sequence-based reagent | MCU siRNA | Invitrogen | #HSS132001-3 |  |
| sequence-based reagent | BAK siRNA | Invitrogen | #HSS184085 |  |
| sequence-based reagent | BAX siRNA | Invitrogen | #HSS141354 |  |
| sequence-based reagent | BOK siRNA | Invitrogen | #HSS141392 |  |
| sequence-based reagent | MET siRNA |  |  | 5′CCAUUUCAACUGAGUUUGCUGUUAA-3′]  5′UCCAGAAGAUCAGUUUCCUAAUUCA-3′  5′CCGAGGGAAUCAUCAUGAAAGAUUU-3’ |
| peptide, recombinant protein | Hepatocyte growth factor/Scatter factor  (HGF/SF) | Peprotech | #100-39 |  |
| commercial assay or kit | Nucleospin RNA/Protein Kit | Macherey-Nagel | #740933.250 | RNA extraction |
| commercial assay or kit | Fast SYBR Green mix | Applied Biosystems | #4385617 | qPCR |
| commercial assay or kit | ASAT/ALAT colorimetric assay | Biolabo | #92025 |  |
| commercial assay or kit | QuickChange site-directed mutagenesis | Stratagene | #210519 | Mutagenesis |
| chemical compound, drug | Anisomycin | Calbiochem | #176880 | Apoptosis inducer |
| chemical compound, drug | Staurosporine | Sigma | #S-5921 | Apoptosis inducer |
| chemical compound, drug | ABT 737 | Santa Cruz Biotechnology | #SC-207242 | BH3-mimetic |
| chemical compound, drug | zVAD-FMK | Calbiochem | #627610 | Pan-caspase inhibitor |
| chemical compound, drug | Q-VD-OPH | SM Biochemicals | #SMPH001 | Pan-caspase inhibitor |
| chemical compound, drug | FuGENE HD | Promega | #E2311 | Transfection reagent |
| chemical compound, drug | X-tremeGENE 9 DNA | Roche | #06365787001 | Transfection reagent |
| chemical compound, drug | Lipofectamine 2000 | Thermo Fisher Scientific | #11668019 | Transfection reagent |
| software, algorithm | ImageJ software | Open source, National Institutes of Health | RRID:SCR_003070 | Measure of weighted colocalization coefficients with the JACoP plugin using Manders Coefficients |
| software, algorithm | SymPhoTime64 | PicoQuant |  | FRET acquisition |
| software, algorithm | NIS | Nikon | RRID:SCR_014329 | FRET acquisition |
